# Development of Tactile Globe by Additive Manufacturing

**DOI:** 10.1007/978-3-030-58796-3_49

**Published:** 2020-08-10

**Authors:** Yoshinori Teshima, Yohsuke Hosoya, Kazuma Sakai, Tsukasa Nakano, Akiko Tanaka, Toshiaki Aomatsu, Kenji Yamazawa, Yuji Ikegami, Yasunari Watanabe

**Affiliations:** 8 Institute Integriert Studieren, JKU Linz, Linz, Austria; 9 Jack Baskin School of Engineering, UC Santa Cruz, Santa Cruz, CA USA; 10 Dipartimento di Meccanica, Politecnico di Milano, Milan, Italy; 11 Support Centre for Students with Special Needs, Masaryk University Brno, Brno, Czech Republic; 12 Department of Mechanical Science and Engineering, Chiba Institute of Technology, 2-17-1 Tsudanuma, Narashino, Chiba 275-0016 Japan; 13 National Institute of Advanced Industrial Science and Technology (AIST), 1-1-1 Higashi, Tsukuba, Ibaraki 305-8566 Japan; 14 Special Needs Education School for the Visually Impaired, University of Tsukuba, 3-27-6 Mejirodai, Bunkyo, Tokyo 112-0015 Japan; 15 Advanced Manufacturing Team, RIKEN Advanced Science Institute, 2-1 Hirosawa, Wako, Saitama 351-0198 Japan

**Keywords:** Additive manufacturing, Topography data, Globe with exact relief, Tactile 3D model, Tactile teaching material, Tactile learning

## Abstract

To understand geographical positions, globes adapted for tactile learning is needed for people with visual impairments. Therefore, we created three-dimensional (3D) tactile models of the earth for the visually impaired, utilizing the exact topography data obtained by planetary explorations. Additively manufactured 3D models of the earth can impart an exact shape of relief on their spherical surfaces. In this study, we made improvements to existing models to satisfy the requirements of tactile learning. These improvements were the addition of the equator, prime meridian, and two poles to a basis model. Hence, eight types of model were proposed. The equator and the prime meridian were expressed by the belt on four models (i.e., B1, B2, B3, and B4). The height of their belt was pro-vided in four stages. The equator and the prime meridian were expressed by the gutter on four models (i.e., C1, C2, C3, and C4). The width of their gutter was provided in four stages. The north pole was expressed by a cone, while the south pole was expressed by a cylinder. The two poles have a common shape in all of the eight models. Evaluation experiments revealed that the Earth models developed in this study were useful for tactile learning of the visually impaired.

## Introduction

Blind people can recognize various shapes through tactile sensations. For example, a tactile map creation service was offered according to the demands of the visually impaired and their helpers to disseminate the use of tactile maps [1–3]. Moreover, research on the utilization of 3D printers to help the visually impaired were conducted [4–6]. Development of the effective models for tactile learning has been reported [7–10].

**Fig. 1.**
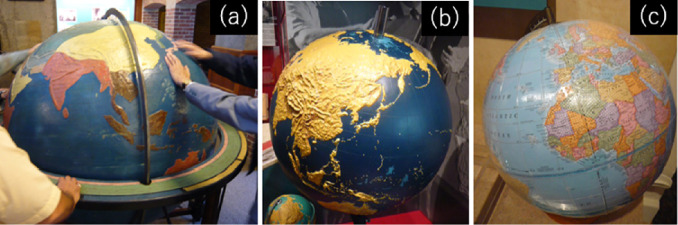
(a) Relief globe (the Perkins School for the Blind), diameter: 135 cm. (b) Relief globe (American Printing House for the Blind), diameter: 76 cm. (c) Tactile and visual Globe (American Printing House for the Blind).

**Fig. 2.**
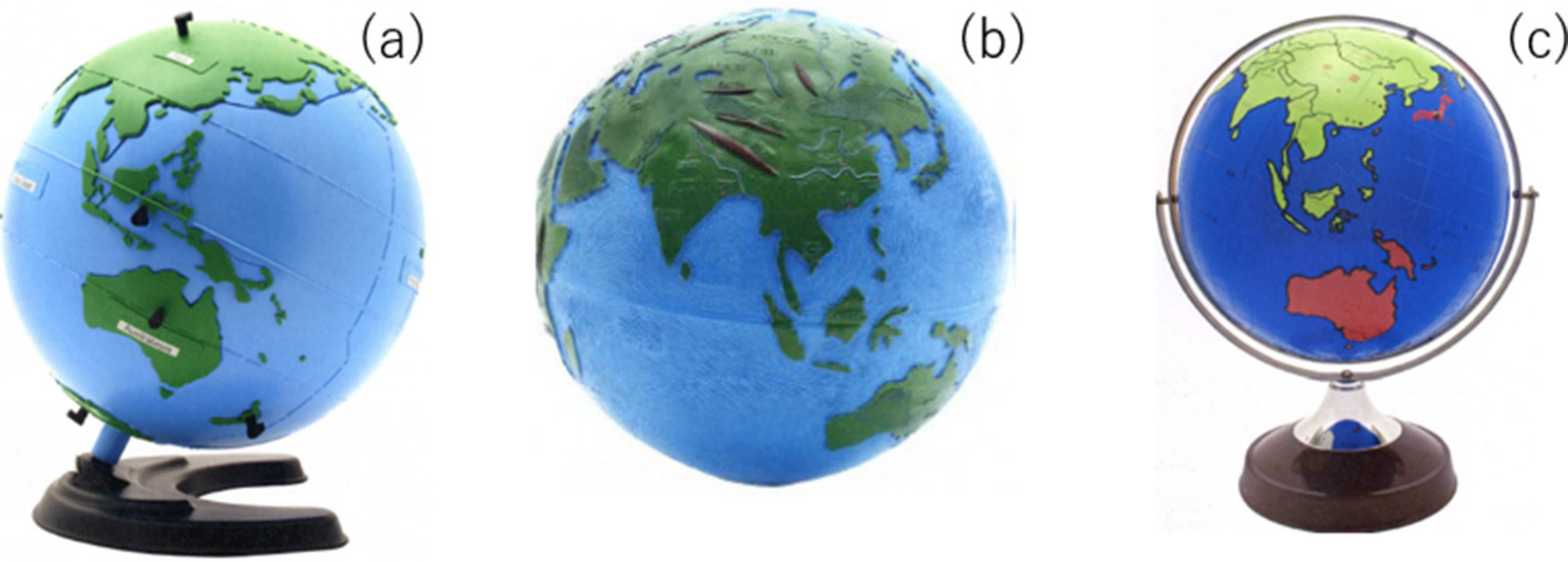
(a) Tactile globe (Royal National Institute of Blind People), diameter: 38 cm. (b) Relief globe (Nippon Charity Kyokai Foundation), diameter: 50 cm. (c) Barrier-free globe (Sun Kougei Inc.), diameter: 32 cm.

The standard globes for the sighted persons have no undulations on their spherical surfaces, and countries and oceans are color-coded on the surface of such globes. Even globes for the visually impaired have been made, with possibly the oldest three-dimensional relief globe made in the United States, which is kept in the lobby of the Perkins School for the Blind. The diameter of the globe is about 135 cm (Fig. 1a). The American Printing House for the Blind has produced two types of tactile globes: one is a relief globe (Fig. 1b) with a diameter of approximately 76 cm, and the other one is the tactile and visual globe (Fig. 1c), which is a standard globe covered with a tactile overlay. The Royal National Institute of Blind People produced a tactile globe (Fig. 2a) whose diameter is about 38 cm. Nippon Charity Kyokai Foundation made a relief globe (Fig. 2b) with a diameter of about 50 cm, while Sun Kougei Inc. produced a barrier-free globe (Fig. 2c) with a diameter of about 32.

These globes have their own features, which are not covered in the scope of this study. Three relief globes are mentioned previously. It is noteworthy that the globes were manufactured without utilizing the exact topography data.

**Fig. 3.**
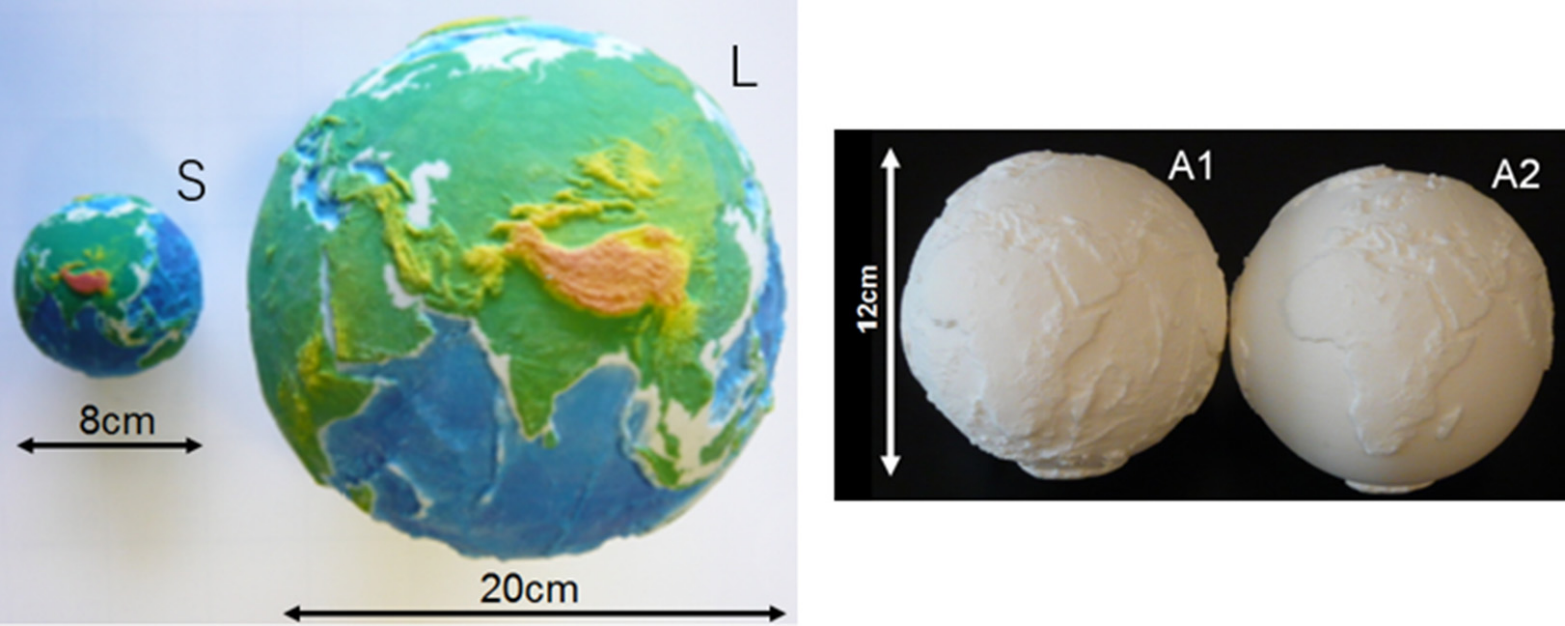
(S, L) Non-modified models. Material: plaster powder (3D printing). (A1) Non-modified model. (A2) Modified model (no relief in the sea). Material: nylon powder (selective laser sinter-ing).

Recently, various additive manufacturing techniques have been developed for globe making. We can 3D print a relief globe that reconstructs the exact topography data by using additive manufacturing. To illustrate such an application, Nakano and Tanaka [11] made a relief globe (Fig. 3, S) and the models of the planets in the solar system. Their models included Venus, Earth, Mars, and Moon. The gradation of colors on the globe corresponds to the elevation of topography.

Teshima et al. [12] developed relief globes that were modified from the model used by Nakano and Tanaka [11] to meet the requirement of tactile learning by the visually impaired. This paper describes in detail both the development of the tactile globe and the evaluation of the level of understanding of the visually impaired.

## Development of Tactile Globes

The diameter of the globe used by Nakano and Tanaka [11] was 8 cm. The first phase of the modification was to change the size of globe. We formed two models with diameters of 20 cm (Fig. 3, L) and 12 cm (Fig. 3, A1). We discussed the suitable size for tactile observation among three models of diameters 20 cm (Fig. 3, L), 12 cm (Fig. 3, A1), and 8 cm (Fig. 3, S) with a blind person. Consequently, the model with a diameter of 12 cm was selected, which is suitable for tactile observation by using both hands. All diameters of the modified models are 12 cm, hereafter.

The second phase of the modification was to delete the relief of the bottom of the sea, on the globe surface, as it is very difficult for the blind to distinguish the land from the sea when such models are used. The abovementioned three models (shown in Fig. 3, S, L, A1) have the relief of the bottom of the sea. In this study, the experimental evaluation of the level of understanding was considered based on the position of the continents. Therefore, we removed the relief of the bottom of the sea (Table 1).

**Table 1.** Model of the earth

Topography data	ETOPO30 from National Snow and Ice Data Center
Real size (radius)	6,378 km
Relief emphasis ratio	50:1
Material	Plaster powder (Fig. 3, S, L), Nylon powder (Fig. 3, A1, A2; Fig. 4)
Diameter of models	8 cm (Fig. 3, S), 20 cm (Fig. 3, L), 12 cm (Fig. 3, A1; Fig. 4)

The procedure for deletion of the relief of the sea is explained in the following steps. First, all the values of the elevation of topography were replaced with −3000 m if the corresponding value was smaller than 0. Next, the updated elevation data were converted into the STL (Stereolithography) format, which is the file format used in the additive manufacturing technique. Finally, the model was constructed (3D printed) by selective laser sintering (the material used was nylon powder). The obtained model (Fig. 3, A2) had a sharp step between the land and the sea, without any relief in the sea.

**Fig. 4.**
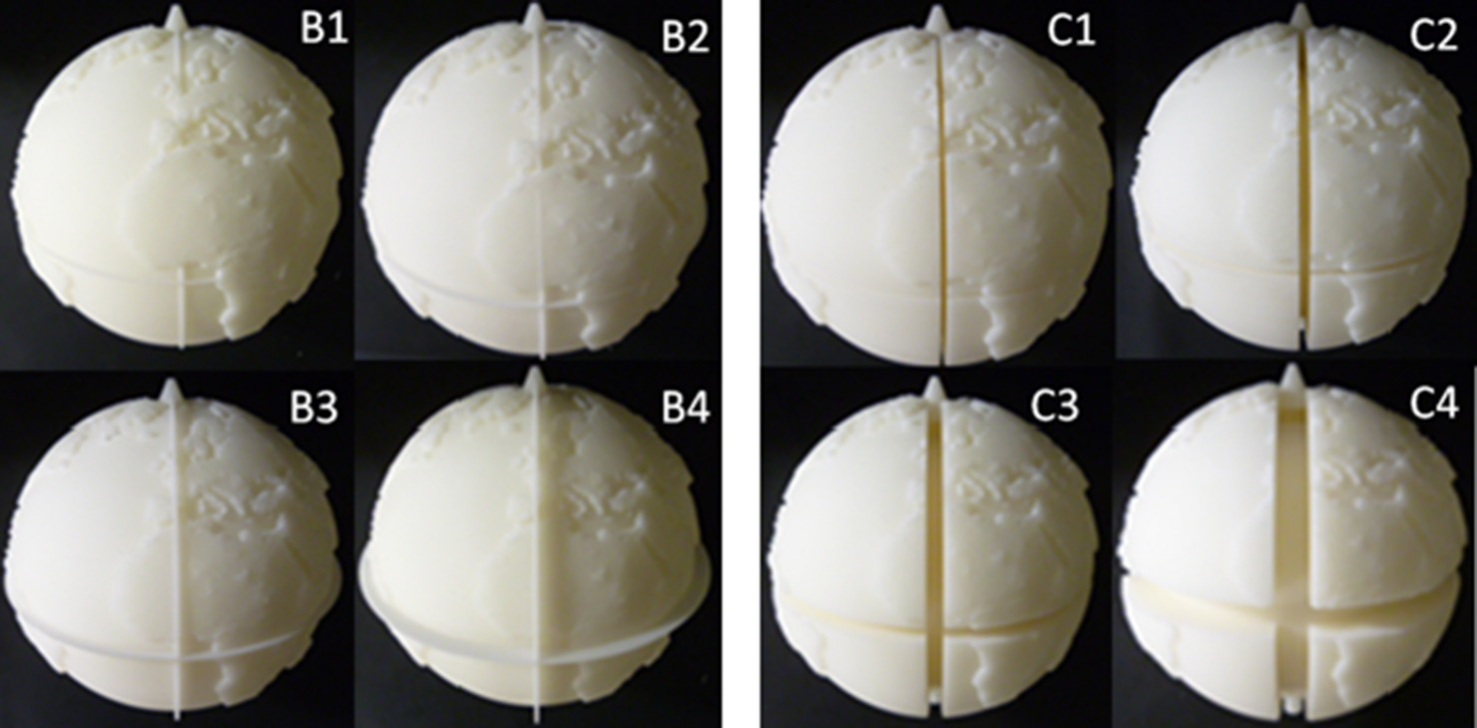
Modified models. Exact topography data were utilized. Height of belt: B1 (1.2 mm), B2 (1.7 mm), B3 (3.3 mm), B4 (6.3 mm). Width of gutter: C1 (1.7 mm), C2 (3.0 mm), C3 (5.3 mm), C4 (10.2 mm). Material: nylon powder (selective laser sintering).

The third and final phase of the modification was to add the equator, prime meridian, and two poles to the model (Fig. 3, A2). The following eight types of model were pro-posed. The equator and the prime meridian were expressed by the belt on models B1, B2, B3, and B4 (Fig. 4). The height of their belt was provided in four stages. The equator and the prime meridian were expressed by the gutter on models C1, C2, C3, and C4 (Fig. 4). The width of their gutter was provided in four stages. The north pole was expressed by a cone, while the south pole was expressed by a cylinder. The two poles have a common shape in all the of eight models.

## Evaluation of the Level of Understanding

Eight subjects who are totally blind took part in our experiments, i.e., the investigation of level of understanding. At the beginning of the experiment, the experimenter explained the positions of both the six continents of the world and Japan to each subject. This advance explanation is shown on model B3.

Next, the subjects were requested to determine the position of each continent or Ja-pan using their index finger, by touching one of the models using both their hands. This examination was performed with four models: B3, C3, A2, and A1.

The experimenter asked each of the subject:

- (Q1) the position of the African continent
- (Q2) to trace the outline of the African continent
- (Q3) the position of the Antarctic continent
- (Q4) the position of the Australian continent, and
- (Q5) the position of Japan.
- The experimenter recorded the answering time per questions.
- Next, the experimenter asked the subjects:
- (Q6) to decide the ranking of the four models (B3, C3, A2, A1) on the basis of their appropriateness for tactile learning
- (Q7) to decide the ranking of the four models (B1, B2, B3, B4) on the basis of their appropriateness for the height of their belts
- (Q8) to decide the ranking of four models (C1, C2, C3, C4) on the basis of their appropriateness for the width of their gutter, and
- (Q9) the most appropriate model in questions (7) and (8).
- The duration of the experiment was about 30 min per subject.

## Results and Discussion

Tables 2, 3, 4, 5 and 6 show the main results of the experiment.

**Table 2.** Answering time on five questions [Q1-Q5] (Average time of eight subjects [s]).

Model	Q1	Q2	Q3	Q4	Q5	Avg.
B3	12.6	12.6	3.0	6.1	10.1	8.9
C3	18.4	18.4	3.4	9.3	4.0	10.7
A2	39.0	39.0	3.5	6.9	4.6	18.6
A1	46.0	46.0	4.6	9.4	25.5	26.3

**Table 3.** Q6: ranking of models (number of subjects).

Model	No. 1	No. 2	No. 3	No. 4
B3	8	0	0	0
C3	0	7	1	0
A2	0	1	7	0
A1	0	0	0	8

**Table 4.** Q7: ranking of belt models (number of subjects).

Model	No. 1	No. 2	No. 3	No. 4
B1	2	1	1	4
B2	1	5	2	0
B3	4	1	3	0
B4	1	1	1	4

**Table 5.** Q8: ranking of gutter models (number of subjects).

Model	No. 1	No. 2	No. 3	No. 4
C1	0	1	4	3
C2	4	2	2	0
C3	4	3	1	0
C4	0	2	1	5

**Table 6.** Q9: belt model vs. gutter model (number of subjects).

Belt model	Gutter model
8	0

Order of usage of the models:

- B3, C3, A2, and A1 for four subjects
- C3, B3, A2, and A1 for four subjects

The last column of Table 2 shows that the best among these four models is model B3 (belt). The second best is model C3 (gutter), followed by model A2 (no relief in the sea), and, finally, model A1 (non-modified model). This order was observed to alter in the observations of Q4 and Q5. This may be attributed to the subjects’ habituation, as the subjects were asked the same questions for every model.

Table 3 shows the ranking of the four models (B3, C3, A2, A1) on the basis of the suitability of tactile learning. All subjects replied that the best model was B3 and the least suitable was A1.

Table 4 shows that model B3 had the most desirable height of 3.3 mm, among all the models. Table 5 shows that models C2 and C3 had the most desirable width of the gutter (3.0 mm and 5.3 mm, respectively).

Table 6 shows that all the subjects answered that the belt models were better than the gutter models. In the case of the gutter model, the width of the gutter leads to the removal of the exact topography. A small width is desirable; however, a gutter with a small width is difficult to recognize. In the case of the belt model, the width of the belt is constant (0.9 mm), while the height of the belt changes.

## Conclusion

The experimental results suggest that the modified globes developed in this study (by additive manufacturing) were found to be useful for tactile learning, as confirmed by the visually impaired.

Now the question is what is the advantage of our method. In our method, the shape of the developed globe can be deformed freely, which can be used to study other geo-graphical features. For example, it will be quite easy to create a relief globe to study the seabed. This globe possesses the relief in the sea but no relief on land, which is the reverse of model A2.

## References

[1] Minatani K, Miesenberger K, Klaus J, Zagler W, Karshmer A (2010). Tactile map automated creation system to enhance the mobility of blind persons—its design concept and evaluation through experiment. Computers Helping People with Special Needs.

[2] Watanabe T, Yamaguchi T, Koda S, Minatani K, Miesenberger K, Fels D, Archambault D, Peňáz P, Zagler W (2014). Tactile map automated creation system using openstreetmap. Computers Helping People with Special Needs.

[3] Watanabe T, Yamaguchi T (2017). Six-and-a-half-year practice of tactile map creation service. Stud. Health Technol. Inform..

[4] Minatani K (2017). An analysis and proposal of 3D printing applications for the visually impaired. Stud. Health Technol. Inform..

[5] Minatani K, Miesenberger K, Kouroupetroglou G (2018). A proposed method for producing embossed dots graphics with a 3D printer. Computers Helping People with Special Needs.

[6] Minatani K, Ahram TZ, Falcão C (2019). Examining visually impaired people’s embossed dots graphics with a 3D printer: physical measurements and tactile observation assessments. Advances in Usability, User Experience and Assistive Technology.

[7] Teshima Y, Miesenberger K, Klaus J, Zagler W, Karshmer A (2010). Three-dimensional tactile models for blind people and recognition of 3D objects by touch: introduction to the special thematic session. Computers Helping People with Special Needs.

[8] Teshima Y, Miesenberger K, Klaus J, Zagler W, Karshmer A (2010). Models of Mathematically Defined Curved Surfaces for Tactile Learning. Computers Helping People with Special Needs.

[9] Teshima Y, Miesenberger K, Klaus J, Zagler W, Karshmer A (2010). Enlarged skeleton models of plankton for tactile teaching. Computers Helping People with Special Needs.

[10] Yamazawa K, Miesenberger K, Karshmer A, Penaz P, Zagler W (2012). Three-dimensional model fabricated by layered manufacturing for visually handicapped persons to trace heart shape. Computers Helping People with Special Needs.

[11] Nakano, T., Tanaka, A.: Making globes of the planets, 3rd Science Frontier Tsukuba, November 2004

[12] Teshima Y, Miesenberger K, Bühler C, Penaz P (2016). Three-dimensional models of earth for tactile learning. Computers Helping People with Special Needs.

